# Modification of Structure, Pasting, and In Vitro Digestion Properties of Glutinous Rice Starch by Different Lactic Acid Bacteria Fermentation

**DOI:** 10.3390/foods14030367

**Published:** 2025-01-23

**Authors:** Dongliang Shao, Jigang Zhang, Tiantian Shao, Yuhui Li, Hongkui He, Yanli Wang, Jintong Ma, Runjie Cao, Anjun Li, Xianfeng Du

**Affiliations:** 1 College of Food and Nutrition, Anhui Agricultural University, Hefei 230036, China; shaodlguoke@163.com; 2 School of Biology, Food and Environment, Hefei University, Hefei 230601, China; zhangjg@hfuu.edu.cn (J.Z.); 18036375235@163.com (T.S.); liyuhui@hfuu.edu.cn (Y.L.); 3 Anhui Risewell Technology Limited Company, Bozhou 236800, China; hehongkui@gujing.com.cn (H.H.); wangyanli2@gujing.com.cn (Y.W.); majintong@gujing.com.cn (J.M.); caorunjie@gujing.com.cn (R.C.)

**Keywords:** starch, glutinous rice, lactic acid bacteria, fermentation modification

## Abstract

This research evaluated the effect of fermentation with *Lactobacillus plantarum* 11122, *Lactobacillus casei* 23184, and *Lactobacillus lactis* 1011 on structure, pasting, and in vitro digestion properties of glutinous rice starch varying in TN and HY genotype, respectively. The results showed that fermentation decreased the weight-average molecular weight and increased the radius of gyration. The short chain was increased by degrading the medium chain (B_2_, DP 24−35) of amorphous in starch, which directly led to the increase of branching degree and rearrangement of the starch chain. LAB fermentation increases the short-range ordered structure, helix structure, and crystallinity by polymerization or interactions of short chains between intermolecular and intramolecular. Furthermore, the pasting characteristic of the fermented starch sample obtained obvious improvement in terms of hydration capacity, including breakdown and setback value. Fermentation facilitated the forming of both slowly digestible starch (17.1–30.79%) and resistant starch (32.3–46.8%) in TN but caused a decline in the content of rapidly digestible starch (25.47–43.6% in TN, 9.36–17.8% in HY). The result of Pearson’s correlation tests and PCA showed the variety of structural and physicochemical of fermentation-treated starch depend highly on the starter culture and starch resources. These results provided new data support for the potential application of modified starch by fermentation with LABs.

## 1. Introduction

Glutinous rice, also named sticky rice, is the hulled seed kernel of the gramineae plant *Oryza sativa* L. var. Glutinosa Matsum. Glutinous rice is one of the favorite traditional Chinese cereals, and the cultivation and consumption of glutinous rice in China has a long history [1]. In general, glutinous rice is used in traditional cuisine to prepare dishes such as Zongzi, Tangyuan, and rice cakes. These dishes are characterized by their rich cultural significance, delicious taste, substantial nutritional value, and outstanding processing qualities, including strong viscosity and good extendibility. Around 59.74~81.14% of glutinous rice is composed of starch, of which 90% is amylopectin. The high content of amylopectin enables glutinous rice to take on excellent physicochemical properties, such as high viscosity and excellent freeze–thaw stability, which endowed it with good processing application. However, there are also some undesirable attributes in glutinous rice starch, such as a lack of ductility (weak water absorption and water retention ability) and low shear resistance, which seriously limit its application within the food industry. Therefore, it is imperative to explore an appropriate modification method in order to improve the techno-functional properties of glutinous rice starch and to satisfy the industrial requirements.

Lactic acid bacteria (LAB) fermentation has been proven to be an effective solution to modify the starch structure and improve the functional characteristics [2,3,4,5]. During fermentation, LAB can produce lactic acid or other organic acids, which, as the acidifying agents, could produce branched chains of amylopectin (first α-1,4 glycosidic bond, then α-1,6 glycosidic bonds) in the amorphous region and convert them into smaller amylose molecules by hydrolysis [6,7]. The main factors that influence the modification of starch include LAB variety, the concentration of acid, fermentation conditions, and substrate. In addition, the hydroxyl groups of starch molecules are esterified by the carboxylic acid group of organic acids to form esterified modified starch [8,9]. LAB secrete various starch-hydrolyzing enzymes, including α-amylase (acts as α,1-4 glycosidic bond in the internal part of amylose or amylopectin), β-amylase (acts as α-1,4 glycosidic bond in external non-reducing ends of starch), amyloglucosidase (acts as α-1,4 glycosidic bond and α-1,6 glycosidic bond), and pullulanase/amylopullulanase (acts as α-1,6 glycosidic bond in branched chains) that play an essential role in modifying the starch structures [4]. Further, there is a complex interaction between organic acid and amylase in the modification of structural characteristics, including coordination and competition. Much research has been conducted to explore the precise regulations of LAB fermentation on the structural modification of starch. Zhang and Liu [10] found that fermentation using *L. plantarum* dy-1 can decompose long side chains of amylopectin as well as increase short chain and amylose content, meanwhile improving compactness, thermal, and shear stability.

Wang and Liu [11] compared the effect of 5 different LAB fermentations on the pasting properties of starch and found that *L. sakei* HSD004 and *L. rhamnosus* HSD005 and their compound starter (in the ratio of 3:1) had a stronger fermentation ability in enhancing the viscosity and reducing the setback and breakdown values of glutinous rice flour. Dinçer and Tekin [2] found that *L sakei* (5.P1.5) and *L. sakei* 8.P1.8 are even capable of fermenting commercial-resistant starch (Hi-maize 260, Novelose 330, and Demirpolat types) as prebiotics to develop functional foods. Wang and Fan [12] found mixed starch (glutinous rice and japonica rice (in the ratio of 2:3)), fermented by *L. acidophilus* HSP001, exhibited optimal pasting properties, solubility, viscoelasticity, and swelling power compared to raw starch. However, LAB fermentation resulted in lower short-range ordered degrees, relative crystallinity, and gelatinization enthalpy. Therefore, the effects of different LAB starter cultures on starches were inconsistent. Due to the different types of enzymes secreted by different LAB during fermentation, the pathy and degree of the enzymatic hydrolysis of substrate starch vary, ultimately leading to different varying degrees of impact on starch structure and properties.

In this research, we will try to explore the effect of fermentation with the *L. lactis* 1011, *L. casei* 23184, and *L. plantarum* 11122 on the structure and physicochemical properties of starch in glutinous rice varying in the TN and HY genotypes, respectively.

## 2. Materials and Methods

### 2.1. Materials

Two different genotypes of glutinous rice were chosen for the investigation of fermentation modification. Huaiyuannuo (HY) is regional to Anhui province, and Tenuo 2072 (TN2072) is regional to Henan province. These two kings of glutinous rice cultivars are widely planted in East China currently. All sample resources were purchased from a local market. The LAB strains *L. lactis* 1011, *L. casei* 23184, and *L. plantarum* 11122 were bought from ACCC (Agricultural Culture Collection of China). The three LAB strains were stored in MRS broth containing 15% glycerol at −80 °C. Each strain was streaked onto MRS agar, incubating at 37 °C for 24 h. One colony was cultivated in 10 mL of MRS broth at 37 °C for 24 h. Prior to vaccinating, the above strains were subcultured again in the MRS broth for 18 h.

### 2.2. Glutinous Rice Pretreatment, Fermentation, and Extraction of Starch

The glutinous rice granule (100 g) was dissolved in 500 mL of distilled water, and the liquid was incubated in a water bath at 40 °C for 4 h. After soaking, the mixture was beaten into a pulp by a strip engine.

Prior to vaccination, each of the 3 LAB strains cultivated to the logarithmic stage was inoculated into the above glutinous rice slurry at a cell concentration of 1 × 10^7^ CFU mL^−1^, respectively. The conical bottle was transferred to an oscillating incubator (HNY-2102C, Honour Instrument Co., Ltd., Tianjing, China) and incubated at 37 °C with a rotation speed of 120 rpm for fermentation, respectively. The fermentation broth (3 mL) was removed to check the pH, LAB numbers, and total titratable acidity when the fermentation was taken place at 0, 6 h, 12 h, 18 h, 24 h, and 30 h, respectively.

After 30 h fermentation, the fermentation broth was centrifuged at 4000 rpm for 10 min to remove the supernatant, and the precipitation was immediately steeped in lye (0.2% NaOH, *w*/*v*) at a ratio of 1:10 (*w*/*v*) for 4 h shake mixing. Then, the mixture was centrifuged (5500 rpm for 10 min), and the upper yellow precipitate was carefully scraped off. The lower precipitate was collected and washed with distilled water. The pH was adjusted to neutral by adding dilute HCl solution into the whished precipitate solution. Finally, after standing precipitation, the starch was collected and dried by vacuum freeze-dried for 48 h. This is the isolation of starch from the powder unfermented glutinous rice (TN2072 and HY) and fermented glutinous rice using LAB (*L. lactis* 1011, *L. casei* 23184, and *L. plantarum* 11122).

### 2.3. pH, Total Titratable Acidity, and Viable Cell Count

During fermentation, the pH value, the total titratable acidity (TTA), and the LAB numbers of the natural unfermented and the different fermentation times (6 h, 12 h, 18 h, 24 h, and 30 h) of glutinous rice slurry were measured, respectively. The pH value of the fermentation broth was measured by using a pH meter (SE-S400-B, Mettler Toledo, Zurich, Switzerland). The TTA was measured according to the method of Wang and Liu [11]. The fermented sample (10 mL) was dissolved in 90 mL of distilled water. The mixture was shaken and filtered through the filter. The filtrate was titrated with 0.1 mol/L sodium hydroxide solution and simultaneously stirred with the electrode of the pH meter until the pH value rose to 8.2. The TTA is calculated by multiplying the volume of sodium hydroxide consumed by 10. The starting LAB strain numbers in the glutinous rice medium during fermentation were determined by viable cell count assays reported Zhang and Hong [13]. Briefly, fermented glutinous rice mixture (1 mL) was added to 9 mL of sterilized saline (0.9% NaCl). The mixture was then diluted properly and spread on the MRS agar plate. After incubating the plate for 48 h at 37 °C, the results were obtained as CFU/mL.

### 2.4. Amylopectin Chain Length Distribution

Referring to the method Shi and Fan [14], the chain length distribution of amylopectin starch was measured with a Dionex ICS5000+HPIC (Thermo Fisher Scientific, Waltham, MA, USA) system equipped with a PAD detector and a CarboPac PA-200 anion-exchange column (4.0 × 250 mm, Dionex, Waltham, MA, USA). The injection volume is 5 μL. The flow rate is 0.4 mL/min. The binary mobile phase consisted of 0.2 M NaOH (A) and 0.2 M NaAc (B) as follows: elution program: 0–10 min, 90% A; 10–30 min, 90–40% A; 30–50 min, 40% A; 50–50.1 min 40–90% A; 50.1–60 min 90% A. Each chain length distribution was calculated using chromeleon 7.2 CDS (Thermo Fisher Scientific Inc., Waltham, MA, USA) by the proportion of each peak area relative to the total peak areas.

### 2.5. Total Molecular Size Distribution of Starch

The weight average molecular weight (Mw) and number average molecular weight (Mn), polydispersity index (PDI, Mw/Mn), and radius of gyration (Rz) of various fractions were detected according to the method Lin and Guo [15]. Starch (5 mg) was thoroughly dissolved in 5 mL DMSO/LiBr (0.5% *w*/*w*) solution by heating conditions at 80 °C for 3 h. The molecular weight distribution of various fractions in starch was determined by the SEC-RI-MALLS system, which is composed of the Ultimate 3000 HPLC (Thermo Fisher Scientific, Waltham, MA, USA) coupled with a Wyatt DAWN HELEOS-II laser scattering detector (He-Ne laser, Wyatt Technology Co., Santa Barbara, CA, USA) and three tandem columns (300 × 8 mm, Shodex OH-pak SB-805, 804 and 803; Showa Denko K.K., Tokyo, Japan). The injection volume was 200 μL. The flow rate was 0.3 mL/min. The dn/dc value was measured to be 0.07 mL/g.

### 2.6. Solid-State 13C Nuclear Magnetic Resonance Spectroscopy

The short-range conformation of starch was investigated using a solid-state 13C -CP/MAS-NMR spectroscopy (Bruker Avance III 400 WB spectrometer, Bruker Inc., Karlsruhe, Germany), coupled with a 4 mm double resonance CP-MAS probe based on the method of Atichokudomchai, Varavinit [16]. The samples were determined at a frequency of 100.62 MHz and scanning times of 1600 ms. The NMR spectra information was obtained and then analyzed and fitted using PeakFit version 4.12 software (Systat Software Inc., San Jose, CA, USA). The relative percentage of the single helix, double helix, and amorphous phase of the starches was calculated according to the methods of Yin and Ma [17]. The amorphous phase (AM) was computed through the ratio of the fitting peak area of the C_4_ (82 ppm) site relative to the total area of the spectrum. The relative percentage of single helix was estimated with the ratio of the fitting peak area of the V-type characteristic peak (94 ppm) relative to the total fitting peak area. The relative double helix content was computed by the ratio of fitting peak areas of C_2,3,5_ (70–75 ppm) relative to the total fitting peak areas.

### 2.7. Branching Degree

The branching degree of amylopectin was measured with Bruker-600 MHz 1H NMR (AVANCE III, Bruker Company, Karlsruhe, Germany) based on the method of [18]. Starch (5 mg) was accurately weighed and transferred into an NMR tube. The dimethyl sulfoxide-D6 solution (DMSO-d6, 0.4 mL) was added, and then ultrasonic vibrated for 1 h in a 100 °C water bath. After obtaining the proton spectrum, the information on chemical shift was analyzed using the MestReNova software (Mnova 15.0.1, Mestrelab Research, Deportivo La Coruna, A Coruña, Spain). The information on the chemical shift of the peak area at 5.4 ppm represents the α-1,4-glucosidic bonds and the peak area at 4.9 ppm corresponds to the α-1,6-glucosidic bonds. Therefore, the branching degree was calculated by computing the ratio of the peak area at 4.9 ppm to the total area of the peak area at 4.9 ppm and 5.4 ppm.

### 2.8. FTIR

According to the method of [17], glutinous rice starch samples of 2 mg were slightly ground with KBr (200 mg) in an agate mortar under infrared lighting heating. The powdered samples were then pressed under vacuum in a metal cylindrical mold of a hydraulic press (YP-2, Shanghai Shanyue Science Instrument Co., Ltd., Shanghai, China) into cylindrical discs (ø: 0.5 mm; H: 3 mm). Before samples were scanned, the spectrum information from pure KBr was collected. The samples with shell shape were scanned using a Fourier transform infrared spectrometer (TENSOR27; Bruker Optics GmBH, Ettlingen, Germany) at the range from 4000 to 400 cm^−1^. The spectrum information subtracted background KBr was then analyzed with OMNIC 9.2 software (Thermo Nicolec Inc, Madison, WI, USA), with the baseline was scaled to the 1200–800 cm^−1^ region, the resolution enhancement factor was set to 1.9, and the half-width was set to 40 cm^−1^ for deconvolution. The ratio of absorption peak intensities at 1047 and 1022 cm^−1^ (R 1047/1022) was used to characterize the short-range ordered degree, and the ratio of those at 995 and 1022 cm^−1^ (R 995/1022) was used to quantify the double helix degree of starch in glutinous rice flour.

### 2.9. X-Ray Diffraction (XRD) Analysis

The starch crystal structures were determined with an X-ray diffractometer (D/Max2550VBt/PC, Rigaku Corporation, Rigaku, Japan) at 40 kV in the range of 5–40° (2θ, 10.0 °/min). The relative crystallinity (RC, %) of starch was calculated by the ratio of crystal peak area to the total diffraction area and analyzed with the jade 6.0 software according to the method of Xu and Man [19].

### 2.10. Pasting Properties

The viscosity of starch during gelatinization was analyzed using a rapid visco analyzer (RVA-5400, Newport Scientific Pty Ltd., Warriewood, Australia). Weigh the starch sample (3.0 g) in an aluminum cylinder, add the deionized water (25.0 mL), and mix well. The sample is placed in RVA to measure the viscosity and the test procedure the concrete method followed the method of Wu and Yu [20]. The gradient program: 50 °C keep for 1 min, heated from 50 °C to 95 °C at 11.25 °C/min, 95 °C kept for 2.5 min, then cooled from 95 °C to 50 °C at 11.25 °C/min, and 50 °C keep for 1.5 min. The rotational speed increased to 960 r/min from the start in 10 s and kept at 160 r/min until the end.

### 2.11. In Vitro Digestibility of Starch

The in vitro starch digestibility was examined according to that described in Englyst and Kingman [21] with minor modifications. Starch samples (100 mg) were dispersed uniformly in sodium acetate buffer (0.1 M, pH 5.2, 10 mL) containing CaCl_2_ (4 mM). The suspension liquid is incubated in a boiling water bath for 30 min, and then, kept in a 37 °C water bath for 30 min. Further, the porcine pancreas α-amylase (2 mg, EC. 3.2.1.1, Sigma) and amyloglucosidase (120 μL, EC. 3.2.1.3, Sigma) were dissolved in a 10 mL sodium acetate buffer (0.1 M, pH 5.2). The enzyme solution was shaken and incubated at 37 °C for 30 min. With mixing between sample liquid and enzyme solution in the ratio of 1:1 (*v*/*v*), the digestion reaction was begun and incubated at 37 °C with shaking at 200 rpm min^−1^. After 20 min and 120 min of digestion, 1 mL of reaction solution was taken with 9 mL of anhydrous ethanol. Next, the samples were centrifuged at 4500 rpm for 5 min. The supernatant is used to determine the glucose content by method of glucose oxidase colorimetric kit (Nanjing Jiancheng Bioengineering Institute, Nanjing, China). The percentages of rapidly digestible starch (RDS), slowly digestible starch (SDS), and resistant starch (RS) were quantified by the following:

$$\begin{array}{c} RDS\left(\%\right)=\left(G20-F\right)\times 0.9/T\times 100,\\ SDS\left(\%\right)=(G120-G20)\times 0.9/T\times 100,\\ RS\left(\%\right)=(1-RDS-SDS)\times 100 \end{array}$$

where F is the glucose content of starch sample at the beginning of the water bath (mg), G20 is the glucose content (mg) of starch sample after 20 min of water bath (mg), G120 is the glucose content (mg) of starch sample after 120 min of water bath, and T is the starch sample weight (mg) used in the experiment.

### 2.12. Statistical Analysis

The data were determined by at least triplicate measurements and results were expressed as means ± standard deviations. T-test was performed to determine the normality of the data. The variance analysis (ANOVA) was performed using SPPS Statistics 19.0 software. The significant differences between the groups were identified with Dunnett multiple comparisons (*p* < 0.05). The Pearson correlation analysis and principal component analysis were conducted with Origin 21.0 software. Characterization of significant differences was conducted at levels of *p* < 0.05.

## 3. Results and Discussion

### 3.1. pH Value, the Total Titratable Acidity, and Viable Cell Counts

**Figure 1:**
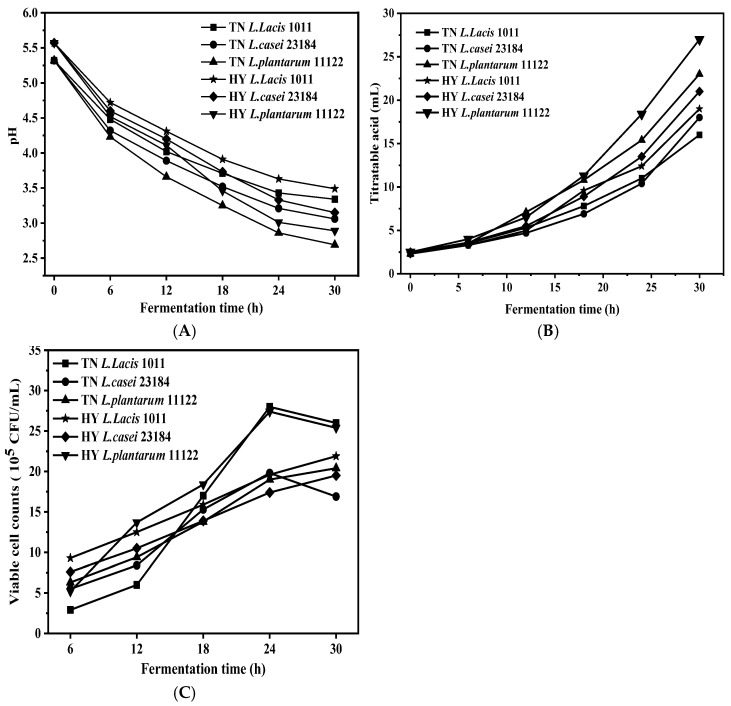
Changes of (**A**) pH value, (**B**) TTA, and (**C**) viable cell number in fermented glutinous rice mixtures.

During LAB fermentation, the viable cell counts, pH, and TTA of glutinous rice broth are shown in Figure 1. At the beginning of fermentation, the action of adding 1 × 10^7^ CFU mL^−1^ of LAB to the substrate was performed, and then the LAB concentration presented an obvious decrease. After 6 h of fermentation, the concentration of *L. plantarum* 11122 in the fermented TN glutinous rice liquid was 5.2 × 10^5^ CFU/mL, and the pH and TTA were 5.3 and 2.5 mL, respectively. With the prolongation of fermentation time, the LAB concentration gradually increased in all fermented glutinous rice flour mixtures, except for TN *L. Lacis* 1011, TN *L. casei* 23184 L, and HY *L. plantarum* 11122 in which LAB concentration appeared in an initial increase and then decrease and reached a peak value by at 24 h. This trend was also found in other LAB fermentations of proso millet flour samples [13]. During fermentation, LAB utilize substrates for continuous colonies, thus generating continuous increases in the concentration of LAB. However, with the advancement of fermentation, the exploitation-competition for substrate resources and the accumulation of major metabolites organic acids changed the survival environment for LAB, leading to a reduction in the total number of bacterial colonies in the later stage. *L. Lacis* had characteristics such as a fast-growing rate, relatively simple metabolism, and separation of decomposition and synthesis metabolism, while *L. plantarum* is thought to adapt to strong adaptability utilizing carbon sources, moving, and preferring aerobic environments [22]. These characteristics ensure that these two species can reproduce and grow quickly, but the nutritional differences between TN and HY make strains prefer to reproduce in TN.

During fermentation, the TTA values of fermented glutinous rice flour mixture gradually increased, whereas the pH values gradually decreased. At the end of fermentation of 30 h, the TTA value of TN and HY glutinous rice flour mixture fermented by *L. Lacis* 1011, *L. casei* 23184, and *L. plantarum* 11122 increased to 16.21, 18.64, 2397, 19.05, 2138, and 27.62 mL, respectively. In parallel, their pH values decreased to 3.34, 3.06, 2.69, 3.49, 3.15, and 2.89, respectively. The increase of TTA and decrease of pH might be owed to the accumulation of major metabolites of LABs, such as L-lactic acid [23]. During the late period of fermentation, the sample fermented by *L. plantarum* 11122 had the highest pH value but the lowest TTA value, indicating that the acid-producing performance of *L. plantarum* 11122 was the strongest of all.

### 3.2. Molecular Weight Distribution

According to the method of Zimm and H. [24], the molecular size distribution of unfermented and fermented glutinous rice starch was calculated, including Mw, Rz, and PDI. As shown in Table 1, the Mw of glutinous rice starch significantly (*p* < 0.05) decreased after LAB fermentation. The Mw of TN starch was 2.77 × 10^5^ kDa and that of fermented samples decreased range from 2.01 × 10^5^~2.43 × 10^5^ kDa. The Mw of the raw HY sample was 2.96 × 10^5^ kDa and decreased range from 1.76 × 10^5^~2.43 × 10^5^ kDa. The decrease in the magnitude of Mw is dependent on the LAB strains, in which the lowest Mw was found by *L. plantarum* 11122, and the highest Mw was found by *L. casei* 23184, indicating that *L. plantarum* 11122 has a stronger capability of modifying Mw. Giraud and Champailler [22] observed that synthesis of the enzyme (high activity of α-amylase) by *L. plantarum* occurred and resulted in hydrolysis of the starch granules during fermentation, leading to the reduction of Mw.

The Rz in the fermented HY glutinous rice starch was significantly higher than that of raw samples, whereas the Rz were similar in unfermented and fermented TN samples, except for *L. plantarum* 11122 fermentations in which the Rz was higher. The PDI of all samples was close, in a small range of 2.03 to 2.21.

Based on the above results, it can be speculated that LAB fermentation reduces the Mw of starch and increases the Rz to varying degrees. The result was consistent with the research of Bian and Chen [25] and Sun and Sun [26]. These results might be mainly attributed to the hydrolysis of the starch molecules by various enzymes (amyloglucosidase, α-amylase) produced by LAB during the fermentation process [27]. The degree of change in starch molecules and radius of gyration is associated with the starter culture and starch source. Li and Wei [28] found *L. plantarum* fermentation is more effective than natural and yeast fermentation and improves the starch quality of buckwheat.

### 3.3. Amylopectin Chain Length Distribution

The distribution of the amylopectin side chain can be categorized into four grades by the degree of polymerization (DP) from small to large: A (DP 6–12), B_1_ (DP 13–24), B_2_ (DP 25–36), and B_3_ (DP ≥ 37) [29]. The percentage of the number of amylopectin side chains in each grade relative to the total number of side chains is shown in Table 1.

In our study, the A, B_1_, B_2_, and B_3_ chains of the TN raw starches were 30.58%, 47.65%, 12%, and 9.74%, respectively. The corresponding chains in HY type were 24.13%, 51.16%, 15.63%, and 8.26%, respectively. Quite a difference in chain-length distribution (CLD) existed between TN and HY varieties. Compared with unfermented samples, the percentage of the B_2_ chain was decreased after fermentation, no matter which type of strain. Fermentation with *L. plantarum* 11122 significantly decreased the percentage of the B_3_ chain and increased that of A chains in both TN and HY, while fermentation with *L. Lacis* 1011 and *L. casei* 23184 L did not cause the change of A chain and B_3_ chain, compared with those of before fermentation. During fermentation, the B_3_ chains in the amorphous region of starch are more vulnerable to organic acid and enzymatic secreted by *L. plantarum* attack, resulting in more short chains (A chains) being formed [30]. No significant difference in the B_1_ chain among various samples. Compared with the native raw sample, (A + B_1_)/(B_2_ + B_3_), the ratio of the short chain (A and B_1_) to the medium-long chain (B_2_ and B_3_) was increased after fermentation.

According to these variations in starch CLDs, we could deduce that the main function of LAB fermentation is to degrade the side chain of medium-long amylopectin into a short branch chain. Prior research reveals that the branching enzymes IIb, hydrolytic enzymes (amylase, isoamylase, and amylopullulanse), and acid released by LAB mainly attacked the amorphous regions of starch, which are mainly composed of medium-long chains [30,31]. Therefore, the medium-long chain was decreased and the short chains were increased.

### 3.4. Solid-State 13C Nuclear Magnetic Resonance Spectroscopy

**Figure 2:**
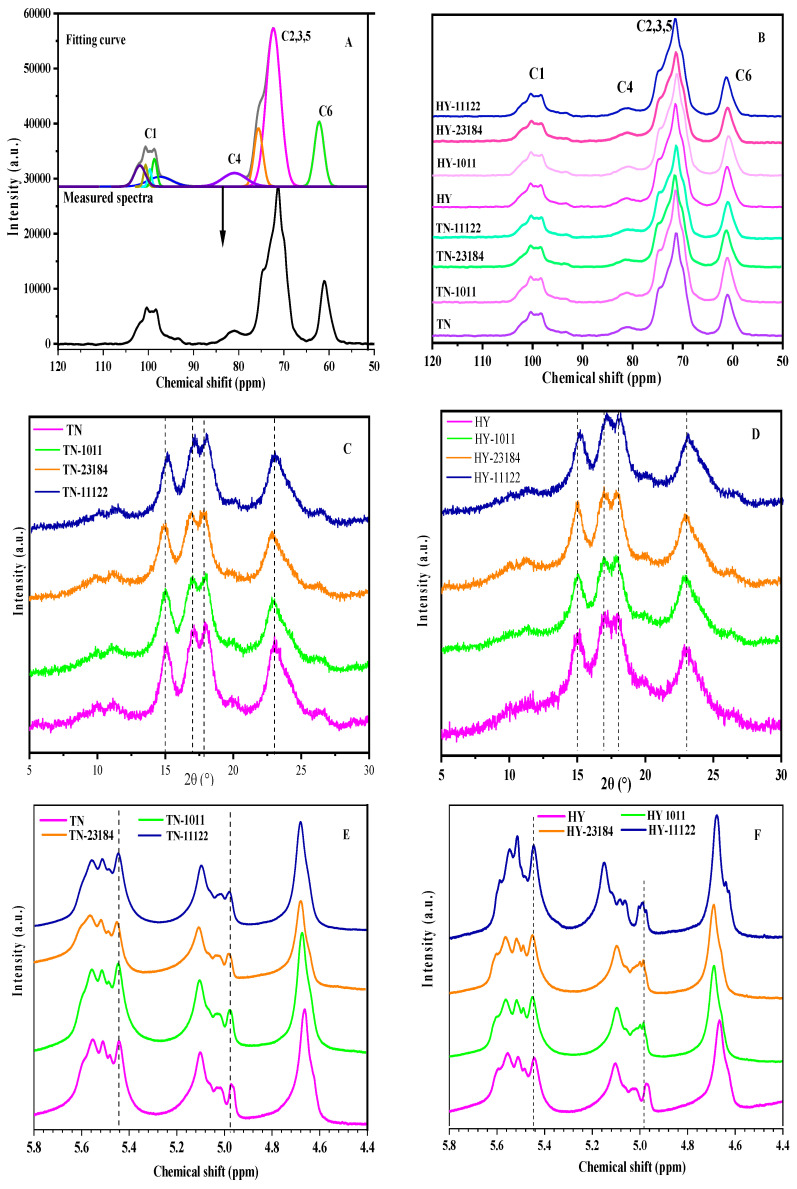
Profiles of ^13^C CP/MAS NMR spectra (**A**,**B**), XRD patterns (**C**,**D**), and ^1^H NMR spectra (**E**,**F**) of glutinous rice starch with different LAB fermentation.

Figure 2 shows the 13C CP/MAS NMR spectra from glutinous rice starches unfermented and fermented with different LAB strains. There were 4 main peak signals located at C_1_ (96–104 ppm), C_4_ (79–83 ppm), C_2,3,5_ (65–78 ppm), and C_6_ (58–64 ppm), respectively. The resonances signal in the C_1_ region has 5 quintuplets, in which two broad shoulders that appeared at 103 and 95 ppm could arise from the amorphous domains. There were triplet peaks in the middle region of C_1_, indicating that both native and LAB fermentation-modified TN and HY glutinous rice starches had typical A-type crystallinity.

The relative proportion of double helixes, single helix, and amorphous components in the total starch structure was calculated by analyzing the 13C CP/MAS NMR spectra according to the Tan and Flanagan [32] method. Table 2 depicts the relative helix contents in various LAB-fermented starch samples. In the HY variety, LAB fermentation with *L. casei* 23184 and *L. plantarum* 11122 significantly increased the double helix content compared with native glutinous rice starches. In the TN variety, no significant difference between the *L. Lacis* 1011 strain fermented starch and the control, but the double helix content at *L. plantarum* 11122 and *L. casei* 23184 fermented starch was obviously higher than the control. Compared with the control, the contents of the single helix of both varieties of glutinous rice fermented with *L. plantarum* 11122 were increased after fermentation. The amorphous structure content of all LAB-fermented starches significantly decreased compared with the native starch. Liu and Xu [33] also found the trend of α-amylase hydrolysis could decrease the amorphous structure and increase the helix of starch. However, different glutinous rice varieties fermented with the different LAB starter cultures exhibited different changes. The *L. plantarum* 11122 strain seems to have stronger abilities in modifying the starch structure. The variation in the structure of *L plantarum* 11122 fermented glutinous rice starch was higher than that of *L. Lacis* 1011 and *L. casei* 23184 fermentation, which may be because more enzymes were released by the *L plantarum* 11122 during the fermentation.

### 3.5. X-Ray Diffraction Pattern and Crystallinity

Figure 2 presents the X-ray diffraction patterns and relative crystallinity at control and fermented glutinous rice with different LAB. All glutinous rice starches showed A-type crystalline structures with characteristic peaks near 15°, 17°, 18°, and 23° (2θ), which is consistent with the result of 13C CP/MAS NMR spectra. The crystallinity of native HY glutinous rice starch was higher than that of TN. After LAB fermentation, the crystallinity of glutinous rice starches has been significantly improved regardless of HY and TN variety. Fermentation by *L. casei* 23184 and *L. plantarum* 11122 resulted in higher crystallinity compared to *L. plantarum* 11122 fermented samples, indicating that the crystalline properties vary with different LAB fermentation strains. In general, the onset of crystallinity was a result of the comprehensive effects of multi-factors, such as chain length distribution, helix arrangement, as well as their interaction [34]. Zhang and Liu [10] reported that *L. plantarum* dy-1 fermentation ameliorates the order degree of the polymeric structure of barley starch, promoting the formation of crystals. The B_2_ chain of the amorphous region in the amylopectin cluster was broken down to produce the short and linear chain by acid and enzymes released by LAB during fermentation. The short linear chain spontaneously could form aggregation and rearrangement, resulting in recrystallization.

### 3.6. Degree of Branching

The 1HNMR spectra of LAB-fermented starches are presented in Figure 2. These outcomes show that the content (11.57%) of native TN starch was analogous to that (12.07%) of the HY starch, while the LAB fermentation-modified glutinous rice starches have a higher branched degree varying from 11.92 to 15.83%. It suggests that the LAB fermentation increased the ratio of α-1,6-glucosidic linkages or decreased the α-1,4-glucosidic linkages in glucose polymers. Guo and Zhu [35] found glucoamylase and branching enzymes could enhance the branching degree by degrading the α-1,4-glycosidic linkages, resulting in the making of amylose tending to amylopectin molecules. We found that fermentation from *L. plantarum* 11122 has a higher branching degree than that of *L. Lacis* 1011 and *L. casei* 23184. Some glucoamylase might be secreted during LAB fermentation. It indicates that *L. plantarum* 11122 provides a more effective enzyme in cleaving the α-1,4-glucosidic bonds to form new branch points.

### 3.7. Molecular Order by FTIR

**Figure 3:**
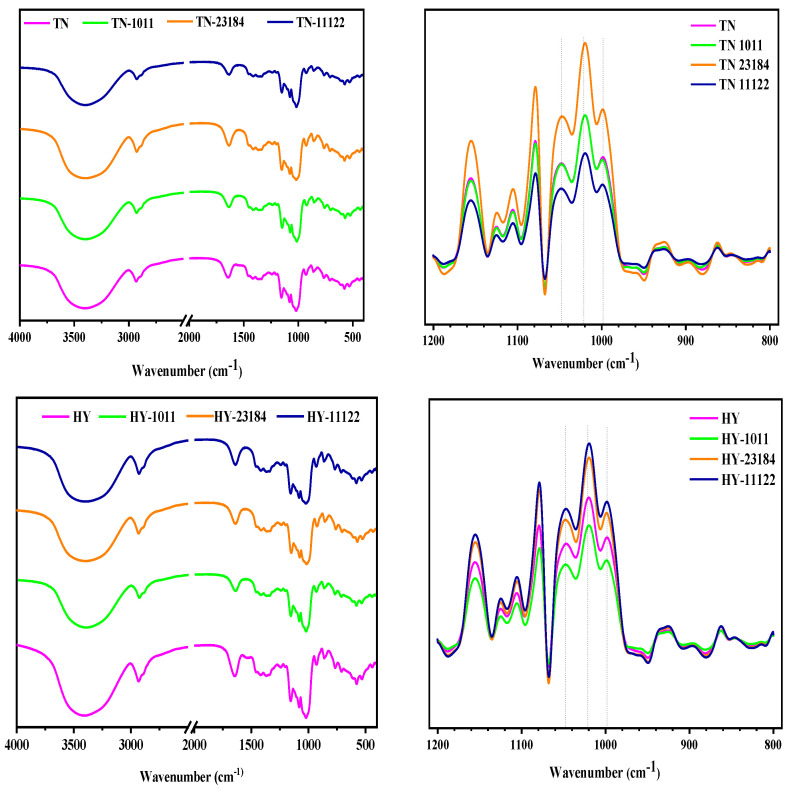
FTIR spectra curve of native and LAB fermented glutinous rice starch.

The original and deconvoluted FTIR spectra of native and LAB-fermented starch are depicted in Figure 3. The fingerprint area at ~995 cm^−1^ and ~1047 cm^−1^ reflect the molecular order and crystallinity of starch polymers, whereas 1022 cm^−1^ is attributed to the amorphous region or disorder phase [36]. Accordingly, the ratio of 1047/1022 cm^−1^ can express the degree of short-range order (DO) in starch, while the degree of double helix (DD) was recognized by computing the ratio of the absorption bands at 995/1022 cm^−1^ [37]. The DO values of starch isolated from fermented glutinous rice were higher than that of unfermented starch except for the HY starches fermented by *L. casei* 23184 in which the DO values were similar to the control (Table 3). Thus, the modified starches by LAB fermentation possessed higher DO than the native starch. Furthermore, it could be clearly seen that the DD values were markedly increased compared with native starch, especially for fermented samples with *L. plantarum* 11122, suggesting the increase of the double helix structure.

By taking into consideration the short-range conformation obtained 13C-NMR and CLD by HPAEC, as well as the crystallinity observed in XRD, it was thus hypothesized that during fermentation, the medium-long chain in the amorphous region can be degraded into a short chain without causing the disassociation of double helix structure. These short-range disorder chains further polymerize and rearrange into short-range ordered structures by the interaction of hydrogen bonds between molecules and intramolecular. Such an effect was particularly obvious for *L. plantarum* 11122 fermented starch samples.

### 3.8. Pasting Properties

**Figure 4:**
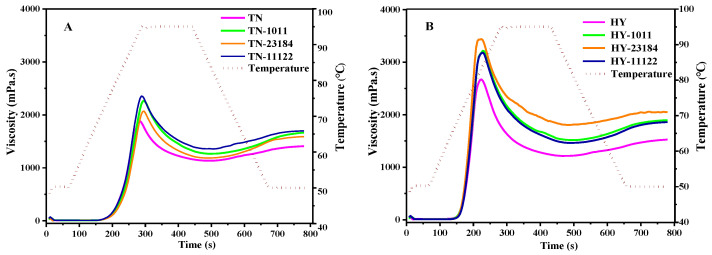
RVA graphs of single LAB strain fermentation on the starch pasting properties of (**A**) TN and (**B**) HY glutinous rice.

The pasting profile of the starches of native and fermented glutinous rice was depicted in Figure 4, with corresponding gelatinization parameters comprising peak viscosity (PV), trough viscosity (TV), breakdown (BD), final viscosity (FV), setback (SB), and gelatinization temperature (GT) summarized in Table 4. The native HY glutinous rice starch exhibited higher PV, TV, and FV viscosity at 2673cp, 1215 cp, and 1526 cp, whereas the TN genotype starch showed lower at 1882 cp, 1135 cp, and 1408 cp, respectively. Relative to native unfermented starch, both PV, TV, and FV exhibited notable increases after the LAB strain fermentation treatment for both varieties (*p* < 0.05), with TN fermented by *L. Lacis* 1011 strain and by *L. plantarum* 11122 and the HY fermented by *L. casei* 23184 experienced a more pronounced effect. The trend of increase after LAB fermentation was in accordance with that reported in a previous study [11,38]. However, the opposite change was found in the barley starch fermented by *L. plantarum*. Zhang and Hong [13] found that the variety of pasting viscosity after fermentation is the opposite between non-waxy samples and waxy samples due to the different content of amylose and amylopectin. The glutinous rice belongs to the waxy samples and has higher amylopectin, which primarily takes responsibility for the development of viscosity and swelling power. During LAB fermentation, the LAB secreted organic acids and enzymes which produced decomposition to starch granules or influenced the ratios of amylose to amylopectin, amorphous to crystalline region, and branching degree, leading to a more compact and ordered structure in hydrogen bonds or higher amylopectin content or starch granules with much swelling [39,40]. Interestingly, the degree of viscosity increase of starch varies significantly among different lactic acid bacteria. For TN starch, the impact of different LAB strains followed the order of *L. Lacis* 1011 > *L. plantarum* 11122 > *L*. For HY starch, it corresponds to *L. casei* 23184 > *L. Lacis* 1011 = *L. plantarum* 11122. Overall, both the starch source and the starter culture are primary factors in determining starch pasting properties.

In addition, the BD is the difference between peak and trough viscosity and reflected paste stability to resist heat disintegration [41]. The SV shows the tendency of starch paste to retrograde [42]. The BD and SB in the fermented glutinous rice starch exhibited an obvious increase to those of native control, except for TN fermented by *L. Lacis* 1011 and *L. casei* 23184 in which SB by *L. Lacis* 1011 was similar to the control while SB by *L. casei* 23184 was lower than control.

### 3.9. Starch Digestibility Characteristics

**Figure 5:**
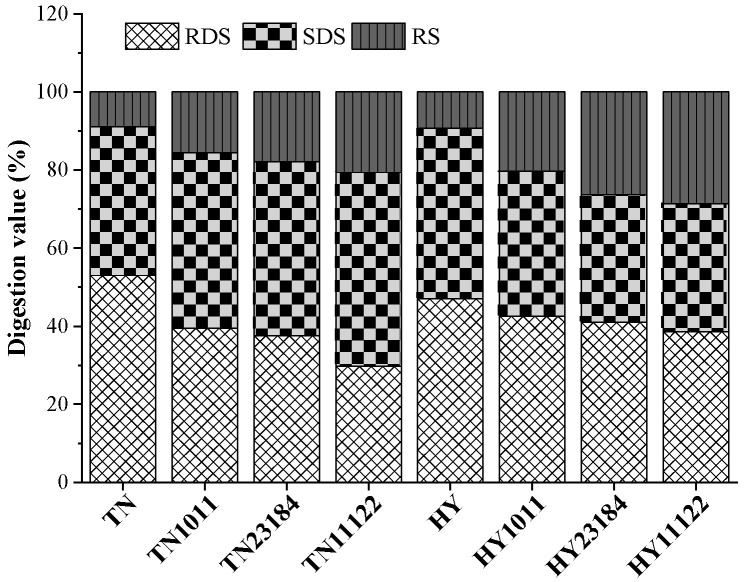
Digestive properties of native and modified glutinous rice starch.

Both varieties of glutinous rice starch contained high RDS and SDS content and low RS contents (Figure 5). Our study found that LAB fermentation reduced RDS content in both varieties, with *L. plantarum* 11122 showing a more apparent reduction rate of 43.96% of TN and 17.87% of HY. This might be attributed to that the LAB fermentation caused the rearrangement of starch molecules to form a more compact and ordered structure, leading to reduced RDS digestibility [43]. Moreover, fermentation significantly increased the SDS content of TN while decreasing it in the HY variety. Specifically, SDS content in TN increased by 18.15%, 17.1%, and 30.79%, while SDS content in TN decreased by 15.05%, 24.97%, and 24.86%, respectively. Furthermore, the RS content in both TE and HY variety exhibited a significant increase in the range of 32.3–46.8%. The above results illustrated that fermentation helps to decrease the glycemic index and promotes gut health [44]. However, the effect of fermentation on the in vitro digestibility of cereal starch depends on the starter culture and starch source. In our research, the TN glutinous rice fermented by *L. plantarum* 11122 had significantly higher SDS and RS content and lower RDS than that fermented by *L. casei* 23184 and *L. Lacis* 1011, which may be attributed to the different amyloglucosidase activities secreted by the strains [45].

### 3.10. Correlation Analysis and Principal Component Analysis

Pearson correlation coefficients between starch molecular structure and pasting/digestion properties were calculated in order to examine the possible relationship and mechanisms of LAB fermentation on starch modification.

**Figure 6:**
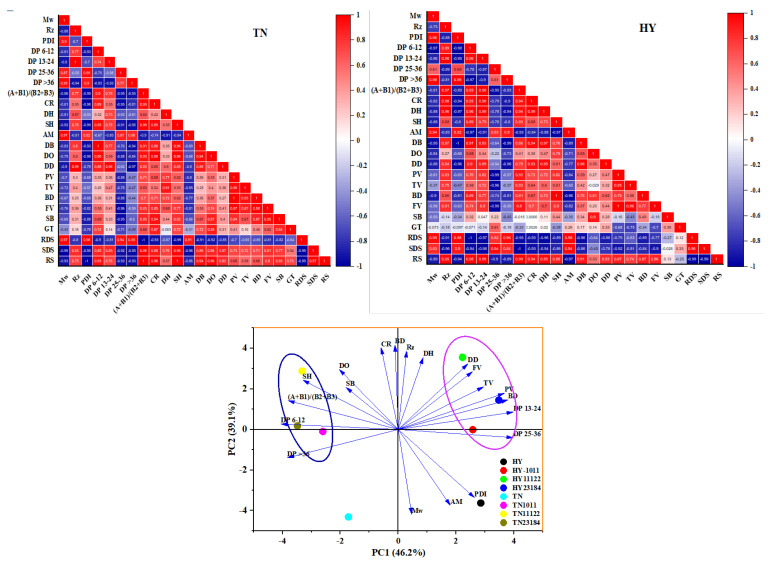
Pearson correlation analysis of structure–property relationships and Principal components analysis of physicochemical properties. Mw: weight average molecular weight, Rz: radius of gyration, PDI: Mw/Mn, DP: degree of polymerization, CR: relative crystallinity, DH: double helices, SH: single helix, AM: amorphous components, DB: Branching Degree, DO: short-range order, DD: degree of double helix, PV: peak viscosity, TV: trough viscosity, BD: breakdown, FV: final viscosity, SB: setback, GT: Gelatinization temperature, RDS: rapidly digestible starch, SDS: Slowly digestible starch, and RS: resistant starch.

As shown in Figure 6, in the pasting characteristics of TN glutinous rice starch, the FV and SB showed an obvious negative correlation with the number of amylopectin medium chains (25 < DP ≤ 36) (r = −0.96, r = −0.95, *p* < 0.05). In HY variety, the PV, TV, and FV showed a remarkable negative correlation with the number of amylopectin medium chains (25 < DP ≤ 36) (r = −0.99, r = −0.96, r = −0.99, *p* < 0.05), while BD value shown a significant positive correlation with single helix content (r = 1, *p* < 0.05) and a significant negative correlation with amorphous content (r = −0.96, *p* < 0.05). This suggests that the number of chains at DP 25-36 oppositely correlated with pasting characteristics.

For the digestion characteristics of TN glutinous rice starch, the RDS content showed a remarkable positive correlation with Mw and PDI (r = 0.97, r = 0.96, *p* < 0.05), but was negatively correlated with the ratio of (A + B_1_)/(B_2_ + B_3_) and single helix content (r = −1, r = −0.99, *p* < 0.05). The SDS showed a significant positive correlation with the ratio of (A + B_1_)/(B_2_ + B_3_) (r = 0.98, r = 0.96, *p* < 0.05) and single helix content, and a significant negative correlation with Mw and amorphous content (r = −0.99, r = −0.96, *p* < 0.05). The RS showed a significant positive correlation with the ratio of (A + B_1_)/(B_2_ + B_3_), crystallinity, single helix content, and the degree of short-range order (r = 1, r = 0.96, r = 1, r = 0.96, *p* < 0.05), but negatively correlated with PDI (r = −1, *p* < 0.05). In HY variety, the RDS showed a significant positive correlation with Mw, PDI, the number of long chains (DP > 36), and amorphous content (r = 0.95, r = 0.98, r = 0.95, r = 0.98, *p* < 0.05), but negatively correlated with the number of short chains (DP 6-16, DP 13-24), the ratio of (A + B_1_)/(B_2_ + B_3_), double helix, and branching degree (r = −1, r = −0.97, r = −0.95, r = −0.95, r = −0.96, *p* < 0.05). The SDS was negatively correlated with Rz, the number of short chain (DP 13-24), and the ratio of (A + B_1_)/(B_2_ + B_3_) (r = −0.96, r = −0.98, r = −1, *p* < 0.05). The RS showed a significant positive correlation with the DP 6-12, 13-24, the ratio of (A + B1)/(B_2_ + B_3_), and DH (r = 0.98, r = 0.99, r = 0.99, r = 0.95, *p* < 0.05) but negatively correlated with AM (r = −0.97, *p* < 0.05). The results suggested that starch molecular size contributes to increasing the RDS and to deducing the SDS and RS, while the ratio of long/short chain contributes to reducing the RDS and increasing the RS, and the helix contributes to reducing the RDS and to increasing RS.

The PCA was used to evaluate the relationship among the different physicochemical parameters of the starch from the two glutinous rice varieties fermented by different LAB. The first (PC1) and second (PC2) principal components accounted for 46.2% and 39.1% of the total variability, respectively. The result showed that there is a long distance between native glutinous rice starch and fermented raw glutinous starch, indicating the obvious changes in physicochemical parameters that occurred after fermentation. The major change of loads between native starch and fermented starches occurred in PC2.

Based on the above results, we hypothesize the possible mechanisms: In the TN variety, the increase of the short chain number and the ratio of (A + B_1_)/(B_2_ + B_3_) formed the helix structure, increased short-range order, and decreased the Mw and increase crystallinity, leading to the increase of BD and SB. In the HY variety, by increasing the medium chain (DP 25-36) and decreasing Mw, it formed more helix structures and less amorphous, leading to an increase in crystallinity and PV, BD FV.

## 4. Conclusions

LAB Fermentation can degrade the B_2_ chain, the ratio of (A + B_1_)/(B_2_ + B_3_), and the branch degree of glutinous rice while increasing the content of short-range ordered and short helix structure. By degrading the amorphous region, the crystallinity of its aggregation structure is improved. In addition, fermentation can improve the swelling stability of glutinous rice starch by increasing its hydration characteristics. Moreover, LAB fermentation can decrease the content of RDS and increase the content of SDS and RS. The *L. plantarum* 11122 has a much stronger ability for starch modification than *L. casei* 23184 and *L. Lacis* 1011 in terms of molecular structure and functional characteristics.

## Figures and Tables

**Table 1:** Weight-average molar mass (MW), radius of gyration (Rz), polydispersity index (PDI), and chain length distribution of amylopectin of starch before and after fermentation.

Samples	Mw	Rz	PDI	Chain Length Distribution (%)				(A + B1)/(B2 + B3)
	×10^5^, kDa	(nm)		A	B1	B2	B3	
TN	2.77 ± 0.10 ^ab^	333.95 ± 0.12 ^bc^	2.16 ± 0.09 ^a^	30.58 ± 0.73 ^b^	47.65 ± 0.52 ^b^	12.00 ± 0.05 ^c^	9.74 ± 0.25 ^a^	3.59 ± 0.03 ^b^
TN1011	2.36 ± 0.07 ^c^	334.13 ± 0.31 ^b^	2.08 ± 0.13 ^a^	30.98 ± 0.32 ^b^	47.73 ± 0.44 ^b^	10.95 ± 0.12 ^d^	9.62 ± 0.12 ^a^	3.82 ± 0.13 ^a^
TN23184	2.43 ± 0.05 ^b^	334.26 ± 0.21 ^b^	2.05 ± 0.08 ^a^	31.87 ± 0.19 ^ab^	47.72 ± 1.35 ^b^	11.03 ± 0.24 ^d^	9.54 ± 0.18 ^ab^	3.86 ± 0.11 ^a^
TN11122	2.01 ± 0.04 ^d^	335.79 ± 0.36 ^a^	2.03 ± 0.14 ^a^	32.12 ± 0.06 ^a^	48.13 ± 0.16 ^b^	10.86 ± 0.18 ^d^	9.34 ± 0.11 ^b^	3.97 ± 0.08 ^a^
HY	2.96 ± 0.11 ^a^	333.58 ± 0.22 ^c^	2.21 ± 0.17 ^a^	24.13 ± 0.33 ^d^	51.16 ± 0.34 ^a^	15.63 ± 0.26 ^a^	8.26 ± 0.09 ^c^	3.18 ± 0.09 ^d^
HY-1011	2.36 ± 0.06 ^c^	334.23 ± 0.34 ^b^	2.16 ± 0.09 ^a^	25.04 ± 0.26 ^cd^	51.23 ± 0.26 ^a^	14.83 ± 0.19 ^b^	8.11 ± 0.11 ^c^	3.32 ± 0.13 ^c^
HY23184	2.43 ± 0.13 ^b^	335.63 ± 0.17 ^a^	2.13 ± 0.01 ^a^	25.33 ± 0.48 ^cd^	51.31 ± 0.12 ^a^	14.28 ± 0.25 ^b^	8.06 ± 0.09 ^cd^	3.43 ± 0.16 ^bc^
HY11122	1.79 ± 0.02 ^d^	335.51 ± 0.06 ^a^	2.07 ± 0.06 ^a^	25.93 ± 0.14 ^c^	51.33 ± 1.09 ^a^	14.73 ± 0.09 ^b^	7.83 ± 0.17 ^d^	3.42 ± 0.14 ^bc^

The values labeled with different letters in each column indicate significant differences among different treatments (*p* < 0.05).

**Table 2:** Crystallinity, short-range conformation, and branching degree of starch samples from native and fermented glutinous rice flours by X-ray diffraction, ^13^C CP/MAS NMR, and ^1^H NMR.

Sample	Crystallinity by XRD (%)	Double Helix by 13C-NMR (%)	Single Helix by 13C-NMR (%)	Amorphous by 13C-NMR (%)	Branching Degree (%)
TN	27.06 ± 0.16 ^e^	62.26 ± 0.36 ^b^	3.21 ± 0.09 ^b^	34.53 ± 0.17 ^ab^	11.53 ± 0.32 ^d^
TN-1011	35.93 ± 0.25 ^b^	64.21 ± 0.21 ^a^	4.36 ± 0.05 ^ab^	31.43 ± 0.23 ^c^	13.21 ± 0.26 ^c^
TN-23184	39.65 ± 0.09 ^a^	62.21 ± 0.42 ^b^	4.88 ± 0.09 ^ab^	32.91 ± 0.35 ^bc^	16.70 ± 0.41 ^b^
TN-11122	39.15 ± 0.31 ^a^	64.55 ± 0.16 ^a^	5.43 ± 0.11 ^a^	30.02 ± 0.29 ^d^	18.05 ± 0.39 ^a^
HY	31.04 ± 0.19 ^d^	62.85 ± 0.09 ^b^	1.23 ± 0.03 ^c^	35.92 ± 0.18 ^a^	12.07 ± 0.55 ^d^
HY-1011	33.01 ± 0.27 ^c^	63.39 ± 0.34 ^ab^	3.31 s ± 0.10 ^b^	33.00 ± 0.36 ^b^	14.25 ± 0.63 ^bc^
HY-23184	38.12 ± 0.04 ^a^	64.27 ± 0.29 ^a^	2.84 ± 0.05 ^bc^	32.89 ± 0.32 ^bc^	16.07 ± 0.39 ^b^
HY-11122	39.54 ± 0.11 ^a^	64.74 ± 0.18 ^a^	3.43 ± 0.03 ^b^	31.83 ± 0.29 ^c^	19.65 ± 0.21 ^a^

The values labeled with different letters in each column indicate significant differences among different treatments (*p* < 0.05).

**Table 3:** Short-range molecular order structure of starch samples isolated from native and fermented Glutinous rice by FTIR.

Sample	DO (1047/1022 cm^−1^)	DD (995/1022 cm^−1^)
TN	0.9316 ± 0.0008 ^d^	0.4895 ± 0.0003 ^e^
TN-1011	0.9883 ± 0.0004 ^b^	0.5000 ± 0.0004 ^d^
TN-23184	1.0377 ± 0.0007 ^a^	0.5179 ± 0.0005 ^d^
TN-11122	1.0328 ± 0.0019 ^a^	0.5778 ± 0.0006 ^b^
HY	0.9600 ± 0.0003 ^c^	0.5509 ± 0.0012 ^c^
HY-1011	0.9876 ± 0.0004 ^b^	0.5597 ± 0.0009 ^c^
HY-23184	0.9614 ± 0.0002 ^c^	0.5984 ± 0.0008 ^b^
HY-11122	0.9954 ± 0.0006 ^ab^	0.6532 ± 0.0003 ^a^

The values labeled with different letters in each column indicate significant differences among different treatments (*p* < 0.05).

**Table 4:** Effects of LAB fermentation on the starch pasting properties of TN and HY glutinous rice.

Sample	PV (cP)	TV (cP)	BD (cP)	FV (cP)	SB (cP)	GT (°C)
TN	1882 ± 43 ^f^	1135 ± 39 ^d^	747 ± 13 ^e^	1408 ± 17 ^e^	273 ± 9 ^c^	76.65 ± 0.13 ^b^
TN-1011	2268 ± 66 ^d^	1265 ± 46 ^c^	1003 ± 16 ^c^	1662 ± 19 ^c^	397 ± 8 ^a^	77.4 ± 0.22 ^ab^
TN-23184	2070 ± 39 ^e^	1168 ± 25 ^d^	884 ± 14 ^d^	1590 ± 25 ^cd^	404 ± 11 ^a^	78.38 ± 0.19 ^a^
TN-11122	2143 ± 51 ^de^	1231 ± 23 ^c^	912 ± 11 ^cd^	1610 ± 29 ^cd^	379 ± 12 ^ab^	77.5 ± 0.23 ^ab^
HY	2673 ± 53 ^c^	1215 ± 31 ^c^	1458 ± 19 ^b^	1526 ± 33 ^d^	311 ± 9 ^b^	69.25 ± 0.12 ^c^
HY-1011	3217 ± 62 ^b^	1518 ± 27 ^b^	1699 ± 29 ^a^	1895 ± 16 ^b^	377 ± 7 ^ab^	68.55 ± 0.09 ^d^
HY-23184	3439 ± 49 ^a^	1805 ± 31 ^a^	1634 ± 14 ^a^	2049 ± 14 ^a^	244 ± 12 ^c^	68.55 ± 0.13 ^d^
HY-11122	3182 ± 61 ^b^	1464 ± 26 ^b^	1718 ± 18 ^a^	1859 ± 12 ^b^	395 ± 11 ^a^	69.30 ± 0.17 ^c^

The values labeled with different letters in each column indicate significant differences among different treatments (*p* < 0.05).

## Data Availability

The original contributions presented in this study are included in the article. Further inquiries can be directed to the corresponding authors.

## Abbreviations

The following abbreviations are used in this manuscript:

LAB	Lactic Acid Bacteria
HY	Huaiyuannuo
TN2072	Tenuo 2072
TTA	Total titratable acidity
Mw	Weight average molecular weight
Rz	Radius of gyration
AM	Amorphous phase
RDS	Rapidly digestible starch
SDS	Slowly digestible starch
RS	Resistant starch
